# Towards Patient-Specific Modeling of Coronary Hemodynamics in Healthy and Diseased State

**DOI:** 10.1155/2013/393792

**Published:** 2013-03-04

**Authors:** Arjen van der Horst, Frits L. Boogaard, Marcel van't Veer, Marcel C. M. Rutten, Nico H. J. Pijls, Frans N. van de Vosse

**Affiliations:** ^1^Cardiovascular Biomechanics, Department of Biomedical Engineering, Eindhoven University of Technology, 5600 MB Eindhoven, The Netherlands; ^2^Department of Cardiology, Catharina Hospital, 5602 ZA Eindhoven, The Netherlands

## Abstract

A model describing the primary relations between the cardiac muscle and coronary circulation might be useful for interpreting coronary hemodynamics in case multiple types of coronary circulatory disease are present. The main contribution of the present study is the coupling of a microstructure-based heart contraction model with a 1D wave propagation model. The 1D representation of the vessels enables patient-specific modeling of the arteries and/or can serve as boundary conditions for detailed 3D models, while the heart model enables the simulation of cardiac disease, with physiology-based parameter changes. Here, the different components of the model are explained and the ability of the model to describe coronary hemodynamics in health and disease is evaluated. Two disease types are modeled: coronary epicardial stenoses and left ventricular hypertrophy with an aortic valve stenosis. In all simulations (healthy and diseased), the dynamics of pressure and flow qualitatively agreed with observations described in literature. We conclude that the model adequately can predict coronary hemodynamics in both normal and diseased state based on patient-specific clinical data.

## 1. Introduction

 Diagnosis of coronary circulatory disease based on coronary pressure and/or flow has been shown to improve the clinical outcome of treatment [1]. The direct measurement of coronary hemodynamics, however, is still limited to the large epicardial vessels, which means that microvascular disease can only be determined from proximal measurements using an appropriate model of the vessels and their interaction with the cardiac muscle [2].

Due to the location of the coronary arteries, being embedded into the myocardium, the contraction of the heart influences coronary hemodynamics. This results in the unique feature that blood is mainly supplied during diastole, while coronary epicardial pressure is high in systole. Several mathematical models have been proposed to model the effect of cardiac contraction on the coronary vessels. Downey and Kirk [3] proposed the vascular waterfall mechanism, which explained the increased resistance to blood flow by vascular collapse when the intramyocardial pressure, determined by ventricular cavity pressure, exceeds the lumen pressure. Spaan et al. [4] introduced the intramyocardial pump model, which accounted for the role of vascular compliance. The intramyocardial pressure, determined by the ventricular cavity pressure, served as the extravascular pressure. The time-varying elastance concept [5] was first applied to the coronary circulation by Krams et al. [6], in which flow is impeded due to a varying stiffness of the cardiac wall. Other, more elaborate, models also include the effect of the coronary vessels on the cardiac contraction (e.g., [7]).

One-dimensional (1D) wave propagation models are potentially useful in the interpretation of arterial hemodynamics [2, 8]. They can provide better insight into the effect of combinations of epicardial and microvascular disease on clinically used indices. Application of 1D wave propagation models to the coronary circulation has been reported in several studies. Smith et al. [9] and Huo and Kassab [10] applied 1D models to vessels representing the coronary arterial anatomy. However, in these studies the interaction with the cardiac muscle was not taken into account. The effect of the cardiac contraction was taken into account in the 1D models of Mynard and Nithiarasu [11], where the coronary vessels were loaded with an approximated left ventricular pressure. Recently, as part of an elaborate arterial tree, Reymond et al. [12] coupled a 1D model to a time-varying elastance model to describe the relation between ventricular pressure, volume, and coronary hemodynamics.

The aim of this study was to construct a model of the human cardiovascular system in which clinical data can be incorporated, to enable patient-specific modeling of coronary hemodynamics. The different submodels were chosen such that the model complexity remains minimal, while still enabling the incorporation of both normal and diseased physiologic and geometric clinical data. To represent the heart, the single-fiber contraction model [13, 14] was chosen. This model is based on microstructural material and macrostructural geometrical properties, allowing the simulation of cardiac disease with geometry-based parameter changes. The large vessels are modeled one-dimensionally [15] to enable easy implementation of the geometry of the vessels, whereas the small vessels are represented by lumped elements. 

Here, the different components of the model are explained and the ability of the model to describe both normal and pathological coronary pressure and flow dynamics is evaluated via comparison with observations described in literature. Two disease types are modeled: coronary stenoses located in the epicardial vessels and left ventricular hypertrophy with an aortic valve stenosis, affecting the coronary microvasculature.

## 2. Materials and Methods

 The model consists of three main elements: a heart contraction model, a wave propagation model for the large arteries, and lumped elements to model the coronary and systemic microcirculation. As part of the wave propagation model, Bessems [16] developed an element that can describe the effect of a stenosis on the local hemodynamics. Since the wave propagation model and heart model have already been described in Bessems et al. [15] and Bovendeerd et al. [14], respectively, only a short description of the models is given below. 

### 2.1. Heart Contraction Model

 Similar to Bovendeerd et al. [14], the left ventricle is modeled as a thick-walled sphere, consisting of nested spherical shells. When assuming rotational symmetry and homogeneity of mechanical load, the relation between tissue stress and ventricular pressure (*p*_lv_), cavity volume (*V*_lv_), and wall volume (*V*_*w*_) can be described as:

(1) $$\begin{array}{c} p_{\text{lv}}=\frac{1}{3}\left(\sigma_{f_{m}}-2{\overset{-}{\sigma }}_{r_{m}}\right)\ln\left(1+\frac{V_{w}}{V_{\text{lv}}}\right). \end{array}$$

Here, *σ*_*f*_*m*__ is the fiber stress and ${\overset{-}{\sigma }}_{r_{m}}$ is the radial wall stress at *r*_lv_ = $\bar{r_{\text{lv}}}$, the shell located at one third of the ventricular wall. This representative shell was chosen because the strain at this location is similar to the fiber strain [14]. At this location, assuming incompressibility of the myocardial tissue, the fiber stretch (*λ*_*f*_*m*__) and the radial stretch (*λ*_*r*_*m*__) can be related to the ventricular geometry by [13]:

(2) $$\begin{array}{c} \lambda_{f_{m}}=\frac{l_{s}}{l_{s,0}}={\left(\frac{V_{\text{lv}}+(1/3)V_{w}}{V_{\text{lv},0}+(1/3)V_{w}}\right)}^{1/3},\;\lambda_{r_{m}}=\lambda_{f_{m}}^{-2} \end{array}$$

with *l*_*s*_ the instantaneous sarcomere length and *l*_*s*0_ the sarcomere length at *V*_lv,0_; the cavity volume at zero transmural pressure.

The myofibers are modeled one-dimensionally, exerting only stress in the fiber direction. The fiber stress consists of an active (*σ*_*a*_) and passive (*σ*_*p*_) stress component, where *σ*_*p*_ only depends on the sarcomere length (*l*_*s*_), while *σ*_*a*_ also depends on the sarcomere shortening velocity (*v*_*s*_) and time elapsed since activation (*t*_*a*_):

(3) $$\begin{array}{c} \sigma_{f}=\sigma_{p}\left(l_{s}\right)+\sigma_{a}\left(l_{s},v_{s},t_{a}\right). \end{array}$$

The active stress is modeled according to Kerckhoffs et al. [18], which describes a combination of the contractility (*c*) and three functions:

(4) $$\begin{array}{c} \sigma_{a}\left(l_{s},v_{s},t_{a}\right)=cg_{1}\left(l_{s}\right)g_{2}\left(l_{s},t_{a}\right)g_{3}\left(v_{s}\right). \end{array}$$

*g*_1_ relates the active stress to the sarcomere length and is given by:

(5) $$\begin{array}{c} g_{1}\left(l_{s}\right)=\{\begin{array}{cc} 0 & l_{s}\leq l_{sa0}\\ \sigma_{a0}\tanh^{2}\left(c_{a}\left(l_{s}-l_{sa0}\right)\right) & l_{s}>l_{sa0}, \end{array} \end{array}$$

where it is assumed that the fibers can only exert stress in tension. *σ*_*a*0_ and *c*_*a*_ are a scaling and curvature parameter, respectively. *l*_*sa*0_ is the sarcomere length at which the stress is zero. The time dependent activation function *g*_2_ is defined as:

(6) g2(ls,ta) ={0ta<0tanh2(tatr)tanh2(tmax⁡−tatd)0≤ta<tmax⁡0ta≥tmax⁡.

Here, *t*_max⁡_ is the activation duration and *t*_*r*_ and *t*_*d*_ are the activation rise and decay time constant, respectively.

The dependency of the active stress on the sarcomere shortening velocity is modeled hyperbolically:

(7) $$\begin{array}{c} g_{3}\left(v_{s}\right)=\frac{v_{s0}-v_{s}}{v_{s0}+c_{v}v_{s}}\;\text{with}\;v_{s}\left(t\right)=-\frac{dl_{s}\left(t\right)}{dt}, \end{array}$$

with *v*_*s*0_ the unloaded shortening velocity and *c*_*v*_ the curvature of the hyperbolic relation.

The passive stress in the fiber (*σ*_*p*_) and radial (*σ*_*r*_) direction are modeled in a similar way:

(8) $$\begin{array}{c} \sigma_{p}\left(l_{s}\right)=\{\begin{array}{cc} 0 & l_{s}\leq l_{s,0}\\ \sigma_{p0}\left(e^{c_{p}(\lambda_{f}-1)}-1\right) & l_{s}>l_{s,0}, \end{array}\\ \sigma_{r}\left(l_{s}\right)=\{\begin{array}{cc} 0 & l_{s}\geq l_{s,0}\\ \sigma_{r0}\left(e^{c_{r}(\lambda_{r}-1)}-1\right) & l_{s}<l_{s,0}. \end{array} \end{array}$$

The passive stress-length relation is determined by the scaling parameters *σ*_*p*0_ and *σ*_*r*0_ and the curvature parameters *c*_*p*_ and *c*_*r*_, respectively.

The intramyocardial pressure (*p*_im_) is used as the extravascular pressure on the coronary circulation and is assumed to be linearly dependent on the radial position in the wall. The shell at *r*_lv_ = $\bar{r_{\text{lv}}}$ is also considered representative for *p*_im_:

(9) $$\begin{array}{c} {\overset{-}{p}}_{\text{im}}=p_{\text{im}}\left(\bar{r_{\text{lv}}}\right)={\overset{-}{\sigma }}_{r_{m}}+\frac{r_{o,\text{lv}}-\;\bar{r_{\text{lv}}}\;}{r_{o,\text{lv}}-r_{i,\text{lv}}}p_{\text{lv}}, \end{array}$$

with *r*_*i*,lv_ and *r*_*o*,lv_ the inner and outer radius of the ventricle, respectively.

#### 2.1.1. Valves

 Since the atrial contraction was not taken into account, the behaviour of the mitral valve was simplified and modeled as an ideal diode, where the pressure gradient over the mitral valve (Δ*p*_mv_) is determined by Ohm's law:

(10) $$\begin{array}{c} \Delta p_{\text{mv}}=Q_{\text{mv}}R_{\text{mv}}, \end{array}$$

with *Q*_mv_ the flow through the mitral valve and *R*_mv_ defined as:

(11) $$\begin{array}{c} R_{\text{mv}}=\{\begin{array}{cc} R_{\text{mv},o} & \text{if}\;\Delta p_{\text{mv}}\geq 0,\\ R_{\text{mv},c} & \text{if}\;\Delta p_{\text{mv}}<0. \end{array} \end{array}$$

*R*_mv,*o*_ and *R*_mv,*c*_ are the resistance of the valve in the open and closed situation, respectively. For the aortic valve the inertia is taken into account and opens due to a positive pressure gradient (Δ*p*_av_) and closes when the flow through the valve (*Q*_av_) becomes negative. The differential equation relating Δ*p*_av_ to *Q*_av_ is defined as:

(12) $$\begin{array}{c} \Delta p_{\text{av}}=L_{\text{av}}\frac{dQ_{\text{av}}}{dt}+R_{\text{av}}Q_{\text{av}}. \end{array}$$

Here, *R*_av_ is the resistance and *L*_av_ the inertia of the valve. *L*_av_ is determined by the cross-sectional area *A*_*v*_, the effective valvular length *l*_av_, and blood density *ρ*; *L*_av_ = *ρl*_av_/*A*_av_. The value of the resistance parameter *R*_av_ is determined by the state of the valve:

(13) $$\begin{array}{c} R_{\text{av}}=\{\begin{array}{cc} R_{\text{av},o} & \text{if}\;Q_{\text{av}}\geq 0,\\ R_{\text{av},c} & \text{if}{\;Q}_{\text{av}}<0. \end{array} \end{array}$$

Here, *R*_av,*o*_ and *R*_av,*c*_ are the resistance of the aortic valve in the open and closed situation, respectively. The values of the different parameters are listed in Table 1.

### 2.2. Wave Propagation Model

**Figure 1:**
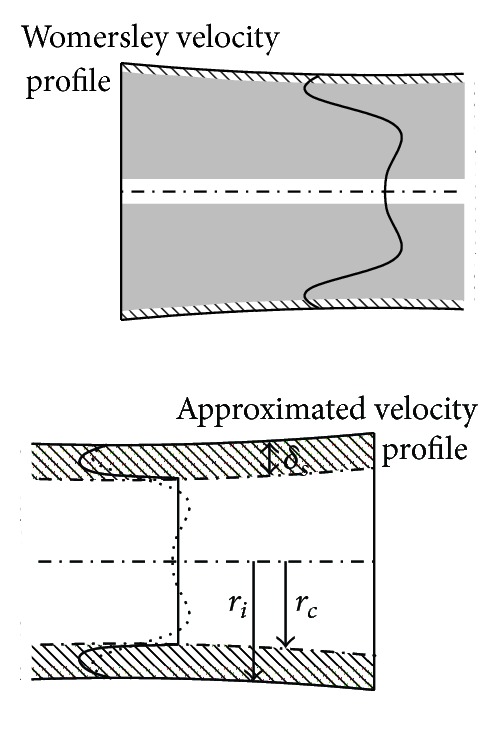
A schematic representation of the exact velocity profile (left) and the approximation (right). *r*_*c*_ is the approximated core radius and *δ*_*s*_ the viscous layer. Adapted from Bessems [16].

 The governing equations describing the one-dimensional propagation of pressure and flow waves of a Newtonian incompressible fluid are derived from the conservation of mass and momentum and were taken from Bessems et al. [15]. The conservation of mass is derived as:

(14) $$\begin{array}{c} \frac{\partial A}{\partial t}+\frac{\partial Q}{\partial z}+\psi =0, \end{array}$$

with *z* and *t* representing the axial direction and time, *A* is the local arterial lumen area, *Q* is the volumetric flow rate, and *ψ* the flow per unit length distributed to small side-branches that are not separately modeled by vascular segments. As described by Bessems et al. [15], an appropriate velocity profile function is assumed that describes the frictional forces and non-linear terms in the balance of momentum equation (see Figure 1):

(15) $$\begin{array}{c} \frac{\partial Q}{\partial t}+\frac{\partial }{\partial z}\left(\delta \frac{Q^{2}}{A}\right)+\frac{A}{\rho }\frac{\partial p}{\partial z}=Af_{z}+\frac{2\pi a}{\rho }\tau_{w}+\frac{\eta }{\rho }\frac{\partial^{2}Q}{\partial z^{2}}. \end{array}$$

Here, *p* is the transmural pressure, *a* is the vessel radius, *f*_*z*_ is the body force, that is, force per unit mass, in the axial direction, and *η* and *ρ* are the dynamic viscosity and density of the fluid, respectively. The wall shear stress is given by:

(16) $$\begin{array}{c} \tau_{w}=-\frac{2\eta }{\left(1-\zeta_{c}\right)a}\frac{Q}{A}+\frac{a}{4}\left(1-\zeta_{c}\right)\frac{\partial p}{\partial z}, \end{array}$$

with $\zeta_{c}={\left(\max[0,(1-\sqrt{2}/\alpha )]\right)}^{2}$ representing the relation between the radius of the inertia dominated core and the thickness of the Stokes layer. *α* is the Womersley parameter according to $\alpha =a\sqrt{2\pi \rho f/\eta }$, with *f* the heart rate. The *ζ*_*c*_ parameter also determines the *δ* parameter of the convective term in (15):

(17) $$\begin{array}{c} \delta =\frac{2-2\zeta_{c}\left(1-\ln\zeta_{c}\right)}{{\left(1-\zeta_{c}\right)}^{2}}. \end{array}$$

#### 2.2.1. Arterial Wall Model

 To solve (14) and (15) a constitutive relation between *p* and *A* is required. In this section we will derive an analytical description of the coronary arterial wall mechanics, with parameters that depend on the geometry of the vessel only and are based on a microstructural constitutive model. The main advantage of this approach is that this analytical model enables easy implementation into the wave propagation equations, while retaining the microstructural properties of the arterial wall.

**Figure 2:**
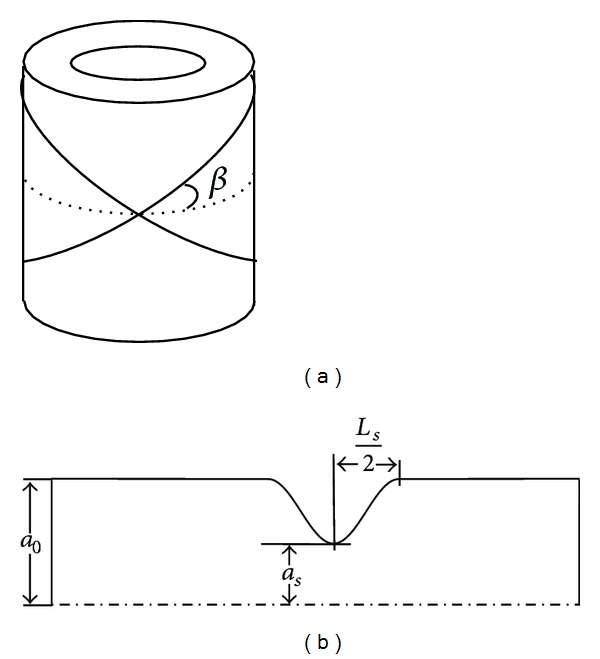
(a) A schematic representation of the model of coronary arterial wall [17]. The fiber orientation is determined by angle *β*. (b) A two-dimensional representation of the stenosis element. *L*_*s*_ is the length of the stenosis, *a*_*s*_ the radius of the vessel at the neck of the stenosis, and *a*_0_ the radius of the vessel proximal to the stenosis. Adapted from Bessems [16].

In van der Horst et al. [20], it was demonstrated that a model based on the two-fiber constitutive model developed by Holzapfel et al. [17] was able to accurately capture the radius-pressure relations of porcine and human coronary arteries. In that model the arterial wall was modeled as a cross-ply of helically wound fibers embedded in a cylinder composed of hyperelastic material. The stress (*σ*)-stretch (*λ*) behaviour is determined by:

(18) $$\begin{array}{c} \sigma =-p_{h}I+G\left(B-I\right)+\sum_{i=1}^{2}\tau_{f}^{i}{\vec{e}}_{f}^{\;i}{\vec{e}}_{f}^{\;i}, \end{array}$$

with *p*_*h*_ the hydrostatic pressure, **I** the unity tensor, *G* the shear modulus, **B** the Finger tensor, and *τ*_*f*_^*i*^ the fiber stress of fiber *i*. ${\vec{e}}_{f}^{\;i}$ is the fiber orientation, which is represented by the angle *β* with the circumference (see Figure 2(a)). It was assumed that in compression no stress can be transmitted by the fibers, with *τ*_*f*_ defined as:

(19) τf=k1λf2(λf2−1)ek2(λf2−1)2 if  λf≥1τf=0 if  λf<1.

Here, *λ*_*f*_ is the collagen fiber stretch and *k*_1_ and *k*_2_ are constants determining the stress-stretch relation of the collagen fibers. For coronary arteries the value of these four parameters have been determined [20]. With removal of the outliers, the median of the four parameters are: *G* = 19.3 kPa, *k*_1_ = 2.01 kPa, *k*_2_ = 5.10, *β*_0_ = 34.6°.

The stress-free geometry is determined by the opening angle parameter and the axial stretch *λ*_*z*_, which are both estimated according to optimization rules explained in van der Horst et al. [20]. The first rule states that at physiological loading the circumferential stress gradients across the wall are minimal. The opening angle parameter is optimized to comply with this rule. The second rule emerges from the finding that the fiber orientation at physiological loading (*β*_phys_) is almost constant for all arteries. Using this rule, *λ*_*z*_ can be directly related to the circumferential stretch at *p* = 13.3 kPa, via two constants: *β*_0_ and *β*_phys_ = 36.4°.

The balance equations resulting from this two-fiber model are solved using numerical integration. Since a numerical integration scheme is also employed to solve (14) and (15), the direct implementation of this two-fiber model will be computationally expensive. Therefore, as an intermediate step, a phenomenological model described by Langewouters et al. [22] is used to analytically relate the compliance (*C*) to the pressure (*p*):

(20) $$\begin{array}{c} C=C_{0}+\frac{C_{1}}{1+{\left(\left(p-p_{m}\right)/p_{w}\right)}^{2}}. \end{array}$$

Here, *C*_0_, *C*_1_, *p*_*m*_, and *p*_*w*_ determine the *C*-*p* relation. To relate this analytical model to the two-fiber model, it is assumed that these four parameters depend on the only clinically measurable quantities: the radius (*a*_*p*_) and wall-thickness (*h*_*p*_) at physiological axial stretch and pressure (*p* = 13.3 kPa). First, the *C*-*p* relation was determined with the two-fiber model (including the optimization rules) for different combinations of *a*_*p*_ and *h*_*p*_. *a*_*p*_ ranged from 0.25 to 3 mm and *h*_*p*_ ranged from 0.025 to 0.3 mm. Then, for each combination of *a*_*p*_ and *h*_*p*_ within the range 0.05 < *κ* < 0.15 (*κ* = *h*_*p*_/*a*_*p*_), the four parameters of the Langewouters model were fitted with the Gauss-Newton algorithm as implemented in Matlab (R2010a, The Mathworks, Natick, MA). Using the *multiple regression* function in Statgraphics (Centurion XVI, statpoint technologies, inc. Warrenton, VA) a polynomial was extracted based on the best *R* ^2^-adjusted value:

(21) C0(ap,hp)=ApC0,1(1+C0,2κ2+C0,3κ), R2=0.999,C1(ap,hp)=ApC1,1(1+C1,2κ2+C1,3κ), R2=0.999,pm(ap,hp)=pm,1+pm,21κ+pm,3κ, R2=0.996,pw(ap,hp)=pw,1+pw,21κ+pw,3κ, R2=0.999.

The parameters (*C*_(0-1,1−3)_, *p*_(*m*,1−3)_, *p*_(*w*,1−3)_) are determined by *A*_*p*_ = *πa*_*p*_^2^ and *κ* and the correlation is good, as indicated by the *R* ^2^ values. With these relations, the compliance of the coronary arteries as function of the instantaneous diameter could be determined and used in the wave propagation model.

For the systemic arterial walls we use a simple linear elastic model:

(22) $$\begin{array}{c} C=\frac{2\pi \left(1-\mu^{2}\right)a_{p}^{3}}{h_{p}E}. \end{array}$$

Here, *E* is the Young's modulus and *μ* is Poisson's ratio. For all systemic arteries, incompressibility is assumed (*μ* = 0.5) and *E* = 0.4 MPa [19].

#### 2.2.2. Stenosis Element

 While one-dimensional theory is suitable to model the pressure and flow waves in relatively straight arteries, it may yield unrealistic results in pathological regions like aneurysms and stenoses. In the derivation of the one-dimensional model it is assumed that variations in the cross-sectional area of the vessels is relatively small, so the radial and circumferential blood velocity is negligibly small with respect to the axial component. Considering that epicardial coronary arteries are prone to the development of stenoses, it is necessary to use a specific element that can be incorporated into to the 1D model.

Bessems [16] developed a 1D stenosis element, based on the semi-empirical relations obtained by Young and Tsai [23, 24] but with an improved contribution of the viscous and unsteady components, to calculate the pressure-drop over an axisymmetric narrowing. The parameters of this model are based on two-dimensional axisymmetrical finite element simulations of stenotic hemodynamics. The axisymmetric stenosis geometry is depicted in Figure 2(b).

From oscillatory flow simulations Bessems [16] derived the following relation for the pressure drop over a stenosis (Δ*p*_*s*_):

(23) Δps=Kv(α)RstQ+ρKt2A02(A0As−1)2|Q|Q+Ku(α)Lu∂Q∂t+Kc(α)Q−.

The parameters *A*_0_ and *A*_*s*_ are the cross-sectional areas of the vessel proximal to and at the neck of the stenosis, respectively. $\overset{-}{Q}$ is the average flow, and *K*_*v*_, *K*_*t*_, *K*_*u*_, and *K*_*c*_ are empirically determined constants obtained by Bessems [16]. They are given by:

(24) $$\begin{array}{c} K_{v}=1+0.053\frac{A_{s}}{A_{0}}\alpha ,\;\;K_{t}=0.95,\\ K_{u}=1.2,\;\;K_{c}=0.0018\alpha^{2}. \end{array}$$

*R*_*st*_ is the resistance and *L*_*u*_ is inertia across the stenosis:

(25) $$\begin{array}{c} R_{st}=\frac{8\eta }{\pi a_{0}^{4}}\int_{L_{s}}\frac{a_{0}^{4}}{a_{s}^{4}\left(z\right)}dz,\;\;L_{u}=\frac{\rho }{\pi a_{0}^{2}}\int_{L_{s}}\frac{a_{0}^{2}}{a_{s}^{2}\left(z\right)}dz. \end{array}$$

*a*_0_ is the radius of the vessel proximal of the stenosis and *a*_*s*_(*z*) the varying radius of the vessel at the site of the stenosis. When assuming that the pressure drop develops linearly over the length of the stenosis, (23) can be written in the following differential form:

(26) ∂Q∂t+KvRstKuLuQ+ρKt2A02KuLu(A0As−1)2|Q|Q  +LsKuLu∂p∂z+KcRstKuLuQ−=0.

The conservation of mass in the stenosis is given by (14) and its compliance is assumed to be negligible.

### 2.3. Lumped Elements

**Figure 3:**
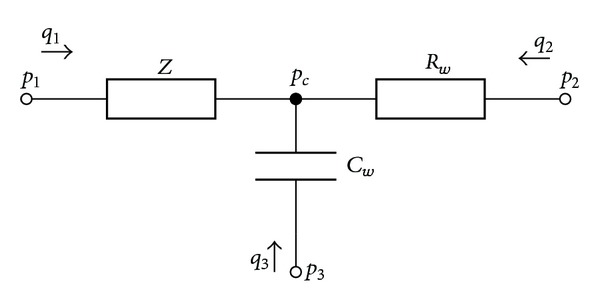
The three-element model with parameters *Z*, *R*_*w*_, and *C*_*w*_.

The contribution of the peripheral vasculature at each end-point of the 1D model is lumped with the three-element model depicted in Figure 3 (see [25]). The relation between the pressure and flow for this model can be written as:

(27) $$\begin{array}{c} {\underline{C}}_{e}\frac{\partial p_{e}}{\partial t}+{\underline{R}}_{e}{\underset{∼}{p}}_{e}={\underset{∼}{q}}_{e}, \end{array}$$

with

(28) C_e=[00000C−C000000−CC0],R_e=[1Z−1Z00−1Z1Z+1Rw0−1Rw0−1Rw01Rw0000],p∼e=[p1pcp2p3],  q∼e=[q10q2q3].

The wave impedance is given by:

(29) $$\begin{array}{c} Z=\sqrt{\frac{\rho }{\overset{-}{A}\;\overset{-}{C}}}, \end{array}$$

with $\overset{-}{A}$ and $\overset{-}{C}$ the average cross-sectional area and compliance of the connecting vessel. The total resistance (*Z* + *R*_*w*_) was determined from the average flow and pressure drop over the element. Finally, *C*_*w*_ is the compliance of the artery defined by a time constant *τ* = *R*_*w*_ *C*_*w*_, with *τ* = 2 s. 

### 2.4. The Complete Model

**Figure 4:**
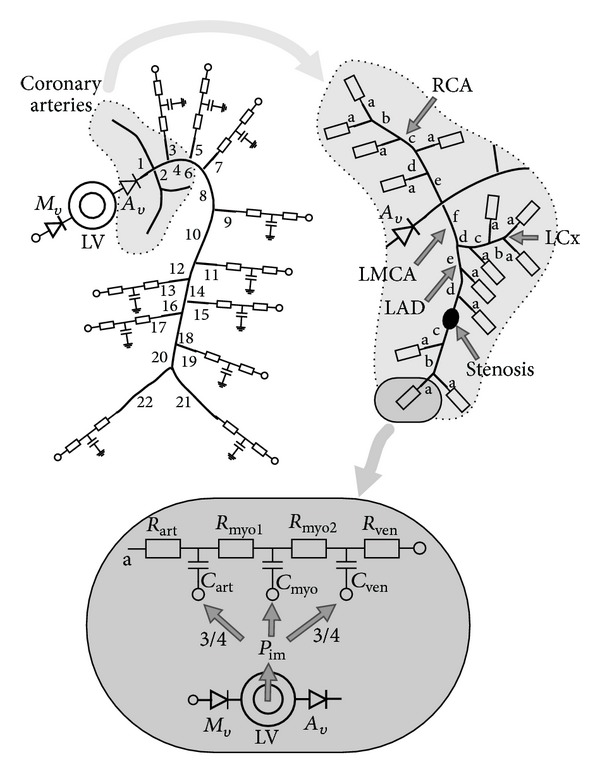
The total model consisting of the left ventricle (LV), with the mitral (*M*_*v*_) and aortic valve (*A*_*v*_), the aorta, and the coronary circulation. The aorta and its main branches are numbered according to Table 2. The LMCA has a length of 5 mm and splits into the LAD and the LCx, with a length 7.5 cm and 6 cm, respectively. Side branches are modeled at intervals of 1.5 cm. Each coronary segment is represented by the characters a–f. The radius of segment a is 1 mm and Murray's law is used to determine the radius of segments b–f. All a-segments are connected to the three-element model representing the coronary microvessels. The intramyocardial pressure (*p*_im_) acts on the three capacitors that represent the vessel compliance. When a stenosis is modeled, it is incorporated into the *c*-segment of the LAD.

 The model of all 1D vessels and lumped elements is shown in Figure 4. The pulmonary venous pressure (1200 Pa ≈ 9 mmHg) serves as the input for the left ventricle (LV) in diastole. The contraction sequence described in Section 2.1 increases the left ventricular pressure (*p*_lv_), closing the mitral valve and, if the pressure exceeds the aortic pressure (*p*_ao_), opening the aortic valve. From the resulting pressure gradient the flow over the aortic valve is calculated using (12) and serves as the input for the aortic wave propagation elements.

To include the effect of the systemic wave reflections, the aorta is modeled with all major side branches, with at each distal end a terminal impedance that is prescribed using the three-element model. The geometrical data are taken from Stergiopulos et al. [19] and are listed in Table 2.

A hypothetical anatomy of the main coronary branches is assumed. The left main coronary artery (LMCA) and right coronary artery (RCA) originate 5 mm distal from the aortic valve. The LMCA splits into the left anterior descending (LAD) and circumflex (LCx) arteries. The LAD has a total length of 7.5 cm with four side branches (representing the diagonal and septal side branches), while the LCx has a length of 6 cm with three side branches (marginal and posteriorlateral branches). The geometry of the RCA is equal to the LAD and it is assumed that it supplies both the left and right ventricle (RV), with a ratio of 0.4. Since the RV is not modeled here, it is assumed that the intramyocardial pressure (*p*_im_) of the RV is smaller by a factor proportional to the ratio of maximum pressure in the two ventricles (*p*_im,rv_ = 0.2  *p*_im,lv_). For all terminal coronary 1D vessels (14 in total) a radius of 1 mm was prescribed and for each bifurcation Murray's law [26] relates the diameter of the parent and daughter vessels by a power of 3. Based on van den Broek et al. [27], it is assumed that for all coronary vessels the wall thickness is equal to 10% of the radius (*κ* = 0.1).

The microvasculature is modeled with three serial three-element models. The total resistance (*R*_*t*_) is determined using Ohm's law by assuming an average pressure of 100 mmHg and prescribing a flow of approximately 20 mL/min through every terminal branch. Following Bovendeerd et al. [14], *R*_*t*_ is distributed over the four resistances according to: *R*_art_ = (7/27)*R*_*t*_, *R*_myo1,2_ = (9/27)*R*_*t*_, and *R*_ven_ = (2/27)*R*_*t*_. The values of the three capacitors are based on measurements by Spaan [28]: *C*_art_ = 0.2 mm^3^ Pa^−1^, *C*_myo_ = 0.53 mm^3^ Pa^−1^, and *C*_ven_ = 0.65 mm^3^ Pa^−1^.   The intramyocardial (*p*_im_) pressure that is generated by the heart contraction model is connected to the three capacitors to model the extravascular pressure on the coronary vessels. Since the circulation is not closed, a constant venous pressure of 700 Pa (±5 mmHg) is prescribed at the output of the model.

To be able to solve the full set of equations, the method of Kroon et al. [29] is used in which the 1D and 0D pressure and flow relations of (14), (15), and (27) are solved fully coupled by writing the differential equations in the same form. The 1D vessels are divided into a number of non-overlapping elements of 2.5 mm and the temporal discretization is performed using the Euler implicit integration scheme. The final set of equations is solved using a direct solver [30], as implemented in the finite element package Sepran (Ingenieursbureau SEPRA, Leidschendam, The Netherlands).

#### 2.4.1. Simulations and Data Analysis

 To test whether the model is able to describe coronary hemodynamics in both normal and pathological situations, three different simulations are performed. The normal, healthy situation is modeled with the parameters listed in Tables 2 and 1. The pressure, flow, and volumes of the heart and aorta obtained with the model are qualitatively compared to similar signals described by Van De Vosse and Stergiopulos [8]. The modeled coronary pressure and flow in the LMCA and RCA are compared to pressure and velocity measurements performed simultaneously in the LMCA and RCA by Hadjiloizou et al. [21]. Two types of pathological situations are modeled: a stenosis in the LAD (see Figure 4) and left ventricular hypertrophy due to an aortic valve stenosis (LVH-AVS).

Dynamic pressure measurements proximal and distal to a mild stenosis (50% diameter, length 2.65 mm) and severe stenosis (70% diameter, length 7.48 mm), performed at the catheterization laboratory of the Catharina Hospital (Eindhoven, The Netherlands), are used to verify the stenosis element. Since the flow through the stenotic vessels was not measured, quantitative comparison is difficult. Therefore, the dynamics of the measured and modeled pressure signals is only compared qualitatively. Since the pressure measurements are performed during hyperaemia, the flow in the model was increased five-fold, by decreasing the coronary microvascular resistances. Furthermore, the clinically most used index to quantify coronary stenoses, fractional flow reserve (FFR), which is defined as the ratio of the pressure distal and proximal to a stenosis, is determined with both the model and the measurements.

The ability of the model to describe coronary hemodynamics when LVH-AVS is present is verified with clinical measurements performed by Hildick-Smith and Shapiro [31]. With transthoracic Doppler echocardiography, they measured the dynamics of flow in the LAD in LVH-AVS patients before and six months after aortic valve replacement (AVR). While the average left ventricular cavity volume was constant before and six months after AVR, the measured average ventricular mass decreased significantly: from 271 to 226 g. The average aortic valve pressure gradient before AVR was 93 mmHg and systemic pressures were normal with a minimum and maximum pressure of 89 and 134 mmHg. The two situations before and after AVR are modeled with ventricular wall volumes based on the measured ventricular wall mass, assuming a mass density of 1.1 kg/L. The contractility and aortic valve resistance are increased such that the pressure gradient across the aortic valve is approximately 93 mmHg, while the average aortic pressure remains normal. The model parameters of the heart contraction model and valves are listed in Table 1. The main features of the dynamics of the modeled flow in the LAD are qualitatively compared to the measurements by Hildick-Smith and Shapiro [31].

The difference between the arterial wall model derived in Section 2.2.1 and a simple linear elastic model (22), with respect to the hemodynamics, is investigated. As it is expected that the difference between the used coronary arterial wall model and a simple linear elastic model is largest in the low pressure range, the difference between the two models is determined both proximal and distal to the severe stenosis described above. The relative differences between the pressure, flow, cross-sectional area, and wall shear stress calculated with the model are quantified with parameter *δ*_*y*_:

(30) $$\begin{array}{c} \delta_{y}=100*\frac{y-y_{\mathrm{lin}}}{\overset{-}{y}/2+{\overset{-}{y}}_{\mathrm{lin}}/2}. \end{array}$$

Here, *y* is the hemodynamic signal obtained with the microstructural-based coronary arterial model and *y*_*lin*⁡_ is the signal obtained with the linear elastic model with a Young's modulus of 1.5 MPa and a Poisson ratio of 0.5.

## 3. Results

### 3.1. Normal Hemodynamics

**Figure 5:**
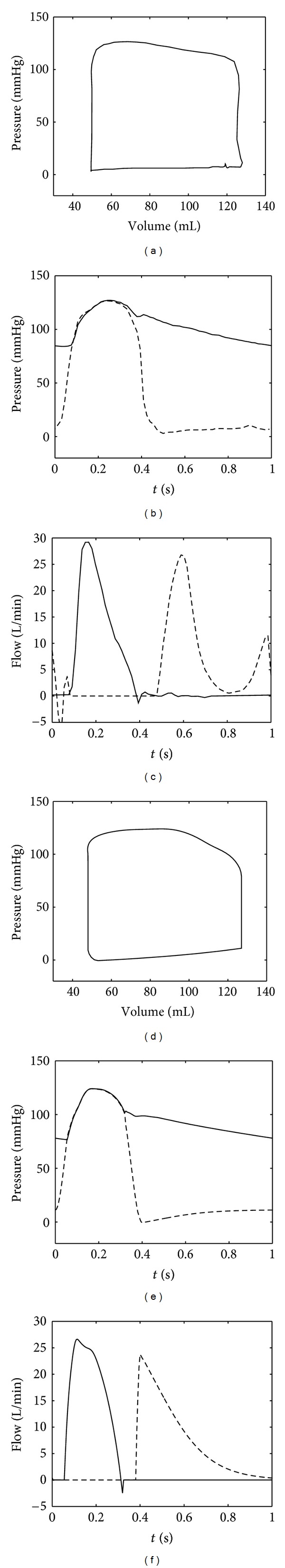
Top: the left ventricular pressure-volume loop (a), the left ventricular pressure (- -) and aortic (-) pressure (b), and the flow through the aortic (-) and mitral (- -) valve (c), adapted from Van De Vosse and Stergiopulos [8] (Figure 3). The pressure-volume loop in (a) is determined from the data in (b) and (c) with the end-systolic volume assumed to be 45 mL. Bottom: similar signals obtained with the model.

Figure 5 shows that the heart and systemic pressures, flows, and volumes obtained with the model, qualitatively agree with values found in literature [8]. With a stroke volume of 70 mL/min, a mean aortic flow of 4.8 L/min, and an aortic mean and pulse pressure of 93 and 47, respectively, the main clinically relevant parameters are within the normal physiological range. At the transition between systole and diastole the effect of the closing of the aortic valve together with a reflection originating from the bifurcation to the iliac arteries is also clearly visible. The dynamics of the different signals are similar, except the time-dependent behaviour of the mitral flow, especially at late diastole (Figures 5(f) and 5(c)). This is obviously due to the lack of the atrial contraction in the model. Another clear difference is that modeled aortic pressure in early systole increases faster than found in Van De Vosse and Stergiopulos [8], which is related to the choice of heart activation function. 

**Figure 6:**
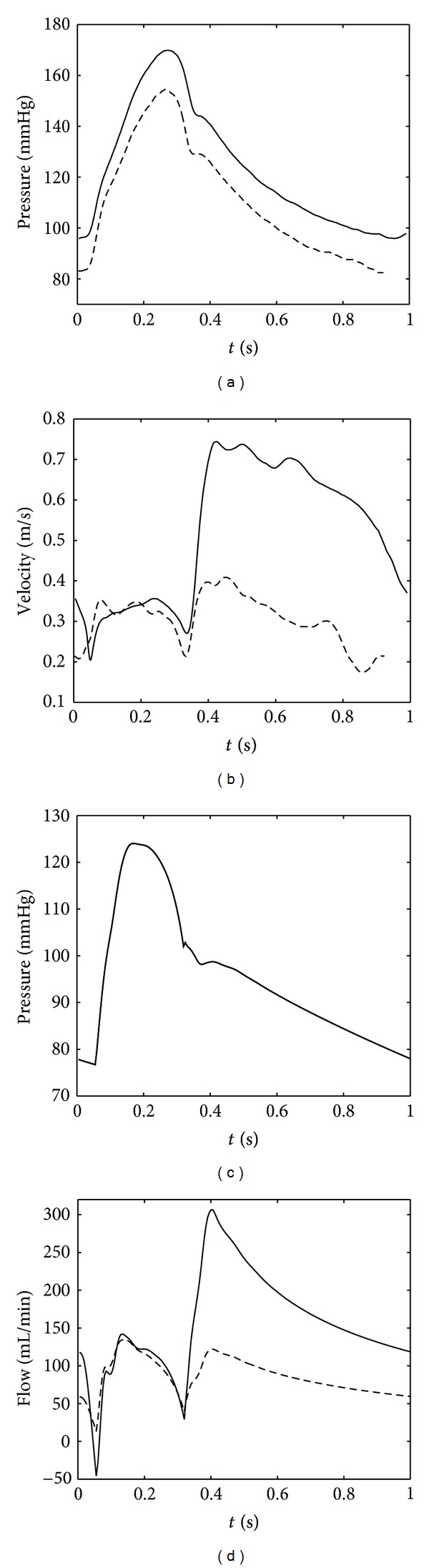
Top: the left main (-) and right (- -) coronary pressure (a) and flow (b), extracted from Hadjiloizou et al. [21] (Figure 1). Bottom: similar signals obtained with the model.

The modeled pressure and fleow in the left main (LMCA) and proximal right (RCA) coronary artery are depicted in Figure 6. The data are compared to pressure and blood velocity measurements acquired simultaneously in a human LMCA and RCA [21]. It should be noted that the mean and pulse pressure measured by Hadjiloizou et al. [21] were relatively high and there was an average offset of approximately 15 mmHg between the pressure measured in the RCA and LMCA (Figure 6(a)). Although these pressures are not considered to be representative for non-diseased vessels, the data do enable the qualitative comparison between the flow velocities in both the LMCA and RCA and the pressure-flow relation. The modeled pressures in the LMCA and RCA were almost identical and are determined by the aortic pressure. The flow in the LMCA was diastolic dominated, with the typical flow impediment during early systole. The ratio of maximum diastolic and systolic flow in the LMCA was 2.1 for both the simulation and measurements. Although it depends to what degree the RCA supplies blood to the left or right ventricle, the flow in the RCA was markedly less dominant in diastole, compared to the LMCA. For the RCA, the ratio of maximum diastolic and systolic flow was 1.2 and 0.9 for the measurements and simulation, respectively. The difference between the LMCA and RCA demonstrates the influence of the intramyocardial pressure on the coronary flow. Besides the pressure in early systole, the main difference between the simulated coronary hemodynamics and the clinical data is that the flow in the early diastole displays a peak in the simulation with subsequently a relatively large decline, whereas this is not the case in the experimental data.

### 3.2. Stenosis

**Figure 7:**
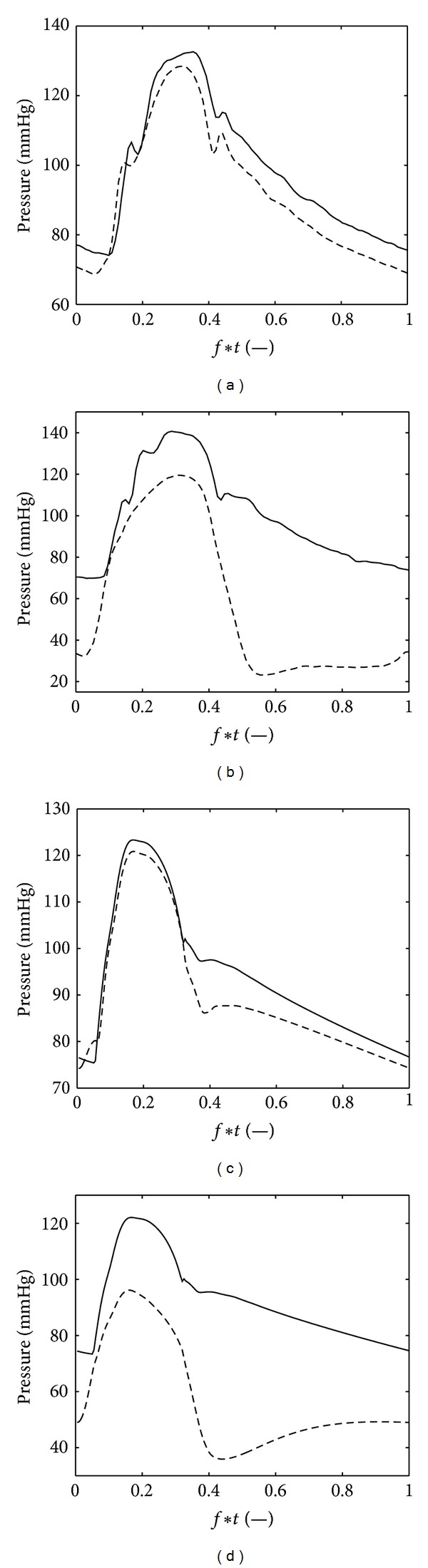
The pressure proximal (-) and distal (- -) to a 50% diameter stenosis with a length of 2.65 mm (a) and (c) and a 70% diameter stenosis with a length of 7.48 mm (b) and (d), measured in human coronary arteries (a) and (b), and determined with the model (c) and (d). Measurements were performed at the Catharina Hospital, Eindhoven, The Netherlands. Written informed consent was given by each patient.

 The pressure measurements depicted in Figures 7(a) and 7(b) demonstrate the effect of a mild stenosis (50% diameter) and severe stenosis (70% diameter), respectively. It is obvious that the pressure gradient was much larger for the severe stenosis, especially in diastole when the flow was highest. The pressures determined with the model showed the same behaviour as the measurements, with the largest pressure gradient in diastole (Figures 7(c) and 7(d)). For the modeled mild stenosis this pressure gradient variation between systole and diastole is larger than in the experimental data. A possible explanation for this discrepancy is a less distinct difference between systolic and diastolic flow in that measurement. The FFR values determined with the measurements were 0.93 and 0.57 for the mild and severe stenosis, respectively, whereas the FFR's determined with the model were 0.96 and 0.61.

### 3.3. Left Ventricular Hypertrophy with an Aortic Valve Stenosis

**Figure 8:**
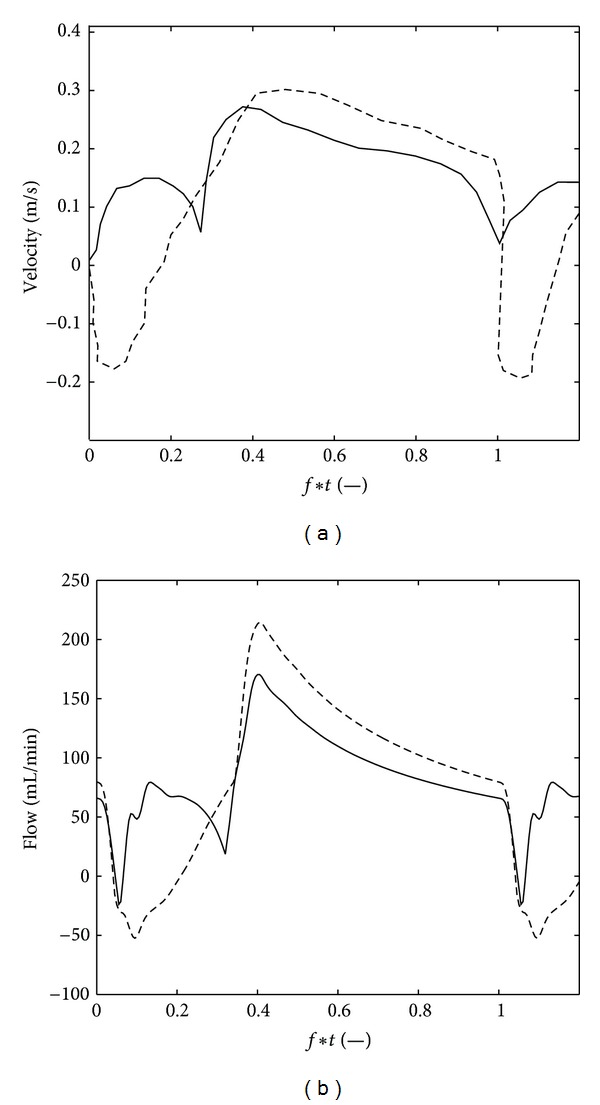
Flow in the LAD of LVH-AVS patients before (- -) and six months after (-) AVR measured in a human LAD with transthoracic Doppler echocardiography by Hildick-Smith and Shapiro [31] (Figures 3(a) and 4(a)) (a) and determined with the model (b).

 In Figure 8 the effect of LVH-AVS on the coronary flow is shown and compared to transthoracic Doppler echocardiography measurements by Hildick-Smith and Shapiro [31], before and six month after AVR. The normal characteristic flow dynamics, with a small positive systolic and large diastole component, was found after AVR, in both the measurements and the simulations. Before the AVR, so when the LVH-AVS is present, the measurements reveal that the positive systolic flow component was replaced by a period of negative flow. The measured maximum diastolic flow decreased slightly after AVR. The ratio of the maximum positive or negative systolic velocity before and after AVR was −1.3, whereas ratio of the maximum diastolic velocity before and after AVR was 1.1. These features were also captured by the model, demonstrating the influence of the increased intramyocardial pressure on the coronary flow dynamics. The ratio of the simulated maximum positive or negative systolic velocity before and after AVR was −0.6, whereas the ratio of the maximum diastolic velocity before and after AVR was 1.3.

### 3.4. Arterial Wall Model

**Figure 9:**
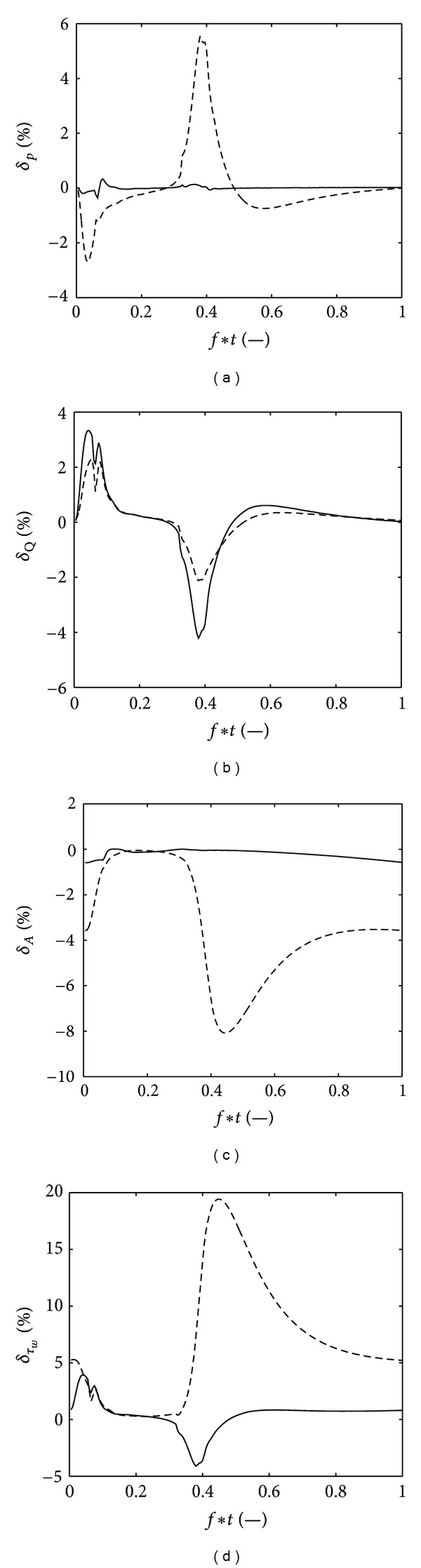
The difference between the results obtained the Langewouters model and linear elastic model, as described in (30). The pressure (*p*), flow (*Q*), cross-sectional (*A*), and wall shear stress (*τ*_*w*_) are shown proximal (-) and distal (- -) to the 70% diameter stenosis.

 The effect of using the arterial wall model proposed by Langewouters et al. [22] compared to a linear elastic model will be most apparent in the low pressure range. Therefore, the difference between the two arterial wall models was determined both proximal and distal to the 70% diameter stenosis. In Figure 9 the relative difference between the two models, as defined by (30), on the pressure (*p*), flow (*Q*), cross-sectional (*A*), compliance (*C*), and wall shear stress (*τ*_*w*_) are shown. Proximal to the stenosis, the difference between the two models was small. While the effect on the pressure (max   *δ*_*p*_ = 5%), flow (max   *δ*_*Q*_ = 2%), and cross-sectional area (max   *δ*_*A*_ = 8%) was rather limited, the wall shear stress changed significantly (max   *δ*_*τ*_*w*__ = 17%). 

## 4. Discussion

 In the present study, previously published models of the heart and vessels have been combined to create a model capable of describing coronary hemodynamics in health and disease. By coupling a heart model to a 1D wave propagation model, the effect of heart disease on both the coronary microvessels and the aortic perfusion pressure could be related to coronary epicardial hemodynamics. With the combination of models, stable solutions were obtained and the waveforms found with the model featured the main characteristics of both systemic and epicardial coronary pressure and flow dynamics. Additionally, by changing a limited amount of parameters, a coronary stenosis and left ventricular hypertrophy with an aortic valve stenosis (LVH-AVS) could be modeled and produced specific hemodynamical features that qualitatively agreed with experimental observations described in literature.

The heart mechanics is governed by the single-fiber contraction model developed by Bovendeerd et al. [14]. The main advantage of this particular model over existing models (e.g., the intramyocardial pump model [4] and the time-varying elastance model [5]) is that it is based on geometric data that can be obtained in the clinic in combination with microstructural properties of the myocardium. This enables the simulation of cardiac disease with physiology-based parameter changes, as was shown by simulating LVH-AVS. Being modeled as a sphere with myofibers oriented in the same direction in each shell, the heart model is a simplified representation of the cardiac muscle. Although the validity of this model should be evaluated for each type of cardiac disease, this simplified representation is also the strength of model, since it is able to produce physiological hemodynamics with a limited amount of parameters. Due to the use of a representative intramyocardial pressure, and average values for the coronary compliances and resistances, the coronary flow in the model should be regarded as a mean flow over the myocardium. It therefore cannot describe radial layer-specific differences in coronary perfusion, which can be clinically relevant in relation to ischemia. Although it will increase the number of parameters, these spatial differences can be incorporated by modeling branches at different layers in the myocardium. Even though the main features of coronary hemodynamics were captured, in future studies it would be interesting to incorporate these branches at different transmural positions to get a more physiologic representative flow distribution in the myocardium. The effect of the deformation of the vessels on its compliance and resistance, especially on the venous side, should then be taken into account as well [32, 33]. From the comparison between the model and literature it was found that the activation model used results in a rise in pressure that is too fast in early systole. An activation function as proposed in van der Hout-van der Jagt et al. [34], in which the early and late part of the activation function can be tuned separately, might prove to resolve this issue. However, this does increase the number of parameters. The contraction of the left atrium was not modeled, which was clearly reflected in the mitral valve flow. This, however, did not result in an unrealistic pressure-volume relation in the left ventricle. The right ventricle was also not incorporated into the model. Therefore, the right ventricular intramyocardial pressure (*p*_im,rv_) was approximated by a factor (0.2) proportional to the left ventricular intramyocardial pressure (*p*_im,lv_). To get a more realistic measure of *p*_im,rv_, the right ventricle can also be modeled with a similar heart contraction model as was demonstrated by Cox et al. [35].

The systemic large epicardial coronary arteries are modeled one-dimensionally, which enables the investigation of the propagating pressure and flow waves as was validated by Bessems et al. [15]. This specific model has the advantage that it is time domain-based and has a velocity profile that approximates the actual Womersley profiles. For the relatively small coronary arteries with Womersley numbers of approximately 2 the velocity profiles are almost similar to the Poiseuille profile, whereas in the aorta there is a phase difference between velocities near the wall and in the central core. This similarity to Womersley profiles is also important when the 1D model is used as the boundary condition for a more detailed 3D model. The choice which branches are lumped or modeled individually, depends on the point of interest and the specific disease that being modeled and the available clinical data. In the model presented here the total aorta and the first part of its side-branches were modeled individually to include its main reflection sites. The coronary arteries with a radius smaller than 1 mm are lumped, since intracoronary measurements are mainly limited to the larger coronary vessels. In future studies, the 1D representation of the coronary vessels also enables the analysis of pressure and flow wave patterns, which have been the subject of recent research [21, 36].

A three-element model was chosen to represent the coronary microvasculature. While this representation did result in physiological coronary hemodynamics, a four-element model with an inertia term [37] might improve the signal, particulary at the large increase in flow during early diastole where the inertia of the blood will play a role. Furthermore, it was found that the parameters of the first three-element model have a large influence on the dynamics of the coronary flow. A proper sensitivity analysis of the model parameters may be helpful in the correct parameter choice for patient-specific modeling.

The compliance of the arterial wall of the coronary vessels was modeled with the analytical model of Langewouters et al. [22]. The parameters of the model were fitted to the model described in van der Horst et al. [20]. For different radii and wall thicknesses an accurate, polynomial description was found for each parameter of the Langewouters model. The main advantage of this approach is that the analytical description enables easy implementation into the model, at low additional computational cost, while the microstructural properties are taken into account. By comparing the pressure and flow waves obtained with this wall model with a linear elastic model, it was found that the differences where very small, even distal to a severe stenosis where the change in compliance are the largest. The wall shear stress, however, did change significantly distal to this stenosis (17%). This might be of clinical interest, since the wall shear stress has been indicated as a factor involved in the development and destabilization of plaques [38].

The stenosis element has been shown to be compatible with the wave propagation elements and agrees qualitatively with pressure measurements from the clinic. The flow in these stenotic vessels was, however, not measured, which makes a proper quantitative comparison impossible. The stenosis is modeled as being smooth and axisymmetric, whereas in clinical practice stenoses are irregular. This might also be the reason why the measured FFR values were lower than the ones obtained with the model. Although 3D modeling [39] is required to investigate to which extent the stenosis element is capable of describing the hemodynamics of irregular stenoses, it is likely that in most cases the stenosis element cannot adequately describe stenoses found in patients. Besides the shape-dependency of the constants of the stenosis element equation, it is also assumed that these constants depend on the heart frequency only, neglecting the contribution of other frequency components. Although Bessems [16] verified the relation with finite element simulations for physiologic flows and found only small differences, this is obviously a limitation of the stenosis model.

The simulated LVH-AVS also qualitatively agreed with the data described in literature, indicating that model is able to capture the global effect of LVH-AVS on coronary flow. However, for a proper verification of the model, simultaneous measurements of left ventricular pressure and volume and coronary pressure and flow should be performed in both non-diseased and LVH-AVS hearts. Furthermore, a number of case studies should also be performed, in which the effect of for different disease types on coronary hemodynamics is measured under well controlled conditions. An isolated beating heart set-up [40] might be a suitable platform for these studies.

The next step in improving the model would be to include autoregulatory mechanisms. The baroreflex mechanism could be included to regulate the heart rate, as was already incorporated into a similar heart contraction model by Cox et al. [35]. Furthermore, by including the coronary autoregulation, the difference between resting and hyperaemic flow can be simulated [41]. This is valuable since it enables the determination of clinical indices based on the difference between resting and hyperaemic hemodynamics, for example, coronary flow reserve [42] or diastolic coronary vascular reserve [43]. This heart contraction model is suitable to include this mechanism, since the work performed by the heart can be used as a parameter in the autoregulation mechanism.

Besides application of this model to enhance the diagnosis in case of combinations of multiple disease types, the model can also be used to investigate the global effect of an intervention, bypass surgery, or collaterals on coronary epicardial hemodynamics. To be able to use the model for patient-specific modeling of the (diseased) coronary circulation, model parameters need to be fitted to hemodynamical measurements. Besides adequate measuring devices for coronary pressure and flow (and lumen area), this will require a proper parameter sensitivity analysis of the model. Due to the number of parameters involved, a Monte Carlo approach as used by Huberts et al. [44] might be suitable to find the most influential parameters. 

## 5. Conclusion

 We constructed a model of the cardiovascular system, in which physiologic and geometric clinical data can be incorporated for patient-specific modeling of coronary hemodynamics. The modeled pressure and flow dynamics are in qualitative agreement with clinical measurements described in literature, especially with respect to the shape details. Although further research is required to improve and verify the model, we conclude that the model adequately can predict coronary hemodynamics in both normal and diseased state based on patient-specific clinical data. 

## Figures and Tables

**Table 1:** The parameters describing the heart, blood, and arterial wall. The values between brackets represent the parameters used to model LVH-AVS before and after AVR, respectively.

Parameter	Value	Unit	Parameter	Value	Unit
*V*_lv, 0_	60 (60, 60)	10^−6^ m^3^	*R*_av, *c*_	1 · 10^12^	Pa s m^−3^
*V*_*w*_	200 (250, 200)	10^−6^ m^3^	*R*_mv, *o*_	4 · 10^6^	Pa s m^−3^
*l*_*s*, 0_	1.9	10^−6^ m	*R*_mv, *c*_	1 · 10^12^	Pa s m^−3^
*l*_*s*, *a*0_	1.5	10^−6^ m	*ρ*	1050	kg m^−3^
*c*	1 (1.4, 1)	—	*η*	0.004	kg m^−1^ s^−1^
*σ*_*a*0_	90	10^3^ Pa	ψ	0	10^−6^ m^3^ s
*c*_*a*_	2.4	10^6^ m	f_z_	0	kg m s^−2^
*t*_*a*_	75	10^−3^ s	C_0, 1_	284	10^−9^ m^2^ Pa^−1^
*t*_*d*_	75	10^−3^ s	C_0, 2_	12.1	—
*t*_max⁡_	0.4	s	C_0, 3_	−3.59	—
*v*_*s*,0_	10	10^−6^ m s^−1^	C_1, 1_	1.09	10^−9^ m^2^ Pa^−1^
*c*_*v*_	1	—	C_1, 2_	34.7	—
*σ*_*p*, 0_	0.9	10^3^ Pa	C_1, 3_	−9.85	—
*c*_*p*_	12	—	p_max⁡, 1_	646	Pa
*σ*_*r*, 0_	0.2	10^3^ Pa	p_max⁡, 2_	−17.0	Pa
*c*_*r*_	9	—	p_max⁡, 3_	15.9	10^3^ Pa
*l*_av_	10	10^−3^ m	p_w, 1_	708	Pa
*A*_av_	679	10^−6^ m^2^	p_w, 2_	−14.8	Pa
*R*_av, *o*_	1 (3 · 10^7^, 1)	Pa s m^−3^	p_w, 3_	12.9	10^3^ Pa

**Table 2:** Geometric and physiological parameters of the arterial vessels. The length (*L*), proximal radius (*a*_*p*_), distal radius (*a*_*d*_), and wall thickness (*h*) of the systemic arteries are based on Stergiopulos et al. [19]. The parameters of the three-element model (*Z*, *R*_*w*_, and *C*_*w*_; see Figure 3) were determined as described in Section 2.3. The numbers of the vessels correspond to the numbers shown in Figure 4.

Nr.	Name	*L*	*r*_ip_	*r*_id_	*h*	*Z*	*R*_*w*_	*C*_*w*_
		mm	mm	mm	mm	MPa s m^−3^	GPa s m^−3^	mm^3^ Pa^−1^
1	ascending aorta A	5.0	14.7	14.7	1.63			
2	ascending aorta B	35	14.7	14.4	1.63			
3	innominate	30	6.20	6.20	0.80	52.2	0.36	4.125
4	aortic arch A	20	11.2	11.2	1.26			
5	left carotid	30	3.70	3.70	0.63	161	1.80	0.835
6	aortic arch B	39	10.7	10.7	1.15			
7	left subclavian	30	4.23	4.23	0.66	118	1.19	1.259
8	thoracic aorta A	52	9.99	9.99	1.10			
9	intercostals	30	2.00	2.00	0.49	659	11.7	0.128
10	thoracic aorta B	104	6.75	6.75	1.00			
11	celiac	30	3.00	3.00	0.64	273	3.40	0.442
12	abdominal aorta A	53	6.10	6.10	0.90			
13	sup. mesenteric	30	4.35	4.35	0.69	112	1.09	1.374
14	abdominal aorta B	10	5.90	5.90	0.80			
15	left renal	30	2.60	2.60	0.53	356	5.28	0.284
16	abdominal aorta C	10	5.90	5.90	0.80			
17	right renal	30	2.60	2.60	0.53	356	5.28	0.284
18	abdominal aorta D	106	5.80	5.48	0.75			
19	inf. mesenteric	30	1.60	1.60	0.43	108	23.1	0.065
20	abdominal aorta E	10	5.20	5.20	0.65			
21	l. common iliac	30	3.68	3.68	0.60	15.9	1.83	0.820
22	r. common iliac	30	3.68	3.68	0.60	15.9	1.83	0.820
